# Small Bowel Gastrointestinal Stromal Tumor (GIST) Presenting With Massive Gastrointestinal Bleeding

**DOI:** 10.7759/cureus.84537

**Published:** 2025-05-21

**Authors:** Mohamed Ahmed, Rasha Saeed, Sarmad Mohammed Salih, Arianne Serrano, Danya Auda

**Affiliations:** 1 Surgery, University of California, Riverside, USA; 2 Occupational Medicine/Environmental Medicine, University of California, Irvine, USA; 3 General Surgery, Östersund General Hospital, Östersund, SWE; 4 Surgery, Oxford University Hospitals NHS Foundation Trust, Oxford, GBR; 5 Colorectal Surgery, AdventHealth Orlando, Orlando, USA; 6 Surgery, AdventHealth Tampa, Tampa, USA; 7 Psychology, University of California, Riverside, USA

**Keywords:** acute gastrointestinal bleed, gastrointestinal stromal tumor (gist), recurrent syncope, small bowel bleeding, small bowel resection

## Abstract

Gastrointestinal stromal tumors (GISTs) of mesenchymal origin may affect any part of the gastrointestinal tract. These tumors are incidentally discovered during endoscopic or radiological investigations, as the clinical manifestations are usually nonspecific. Factors determining prognosis include size, location, number of mitotic figures per high-power field, and tumor rupture. We present the case of an otherwise healthy young man who was brought to our emergency room following a syncopal episode and developed bloody diarrhea en route. The aim is to highlight the need for emergency surgery once diagnosed, as the bleeding is usually massive, recurrent, and life-threatening.

## Introduction

Gastrointestinal stromal tumors (GISTs) are mesenchymal tumors originating from interstitial cells of Cajal within the digestive tract's wall, which play a crucial role in regulating digestive peristaltic and are considered pacemaker cells generating intrinsic electrical rhythmicity and transducing inputs from enteric motor neurons [1]. These tumors are predominantly in the stomach (60%) and less commonly in the small bowel (30%), rectum (5%), and colon (1%) [2]. The clinical manifestations are usually nonspecific, and a small percentage present with acute symptoms such as bleeding requiring an urgent intervention [3,4]. We present acute massive gastrointestinal bleeding secondary to a small bowel GIST requiring emergency surgery.

## Case presentation

**Figure 1:**
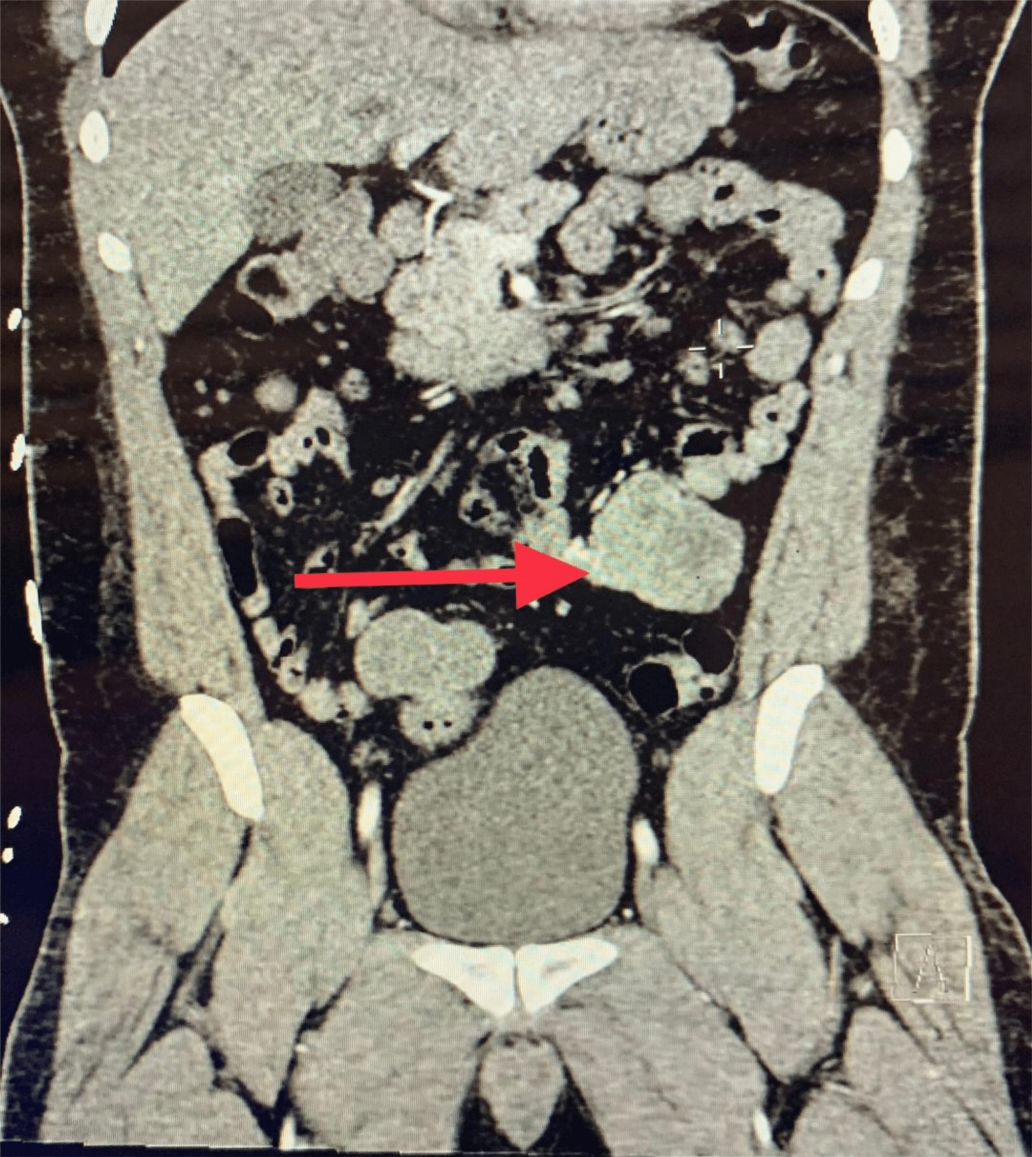
CT (coronal) of the abdomen and pelvis Soft tissue mass associated with small bowel loops in the left lower quadrant (red arrow) CT: computed tomography

**Figure 2:**
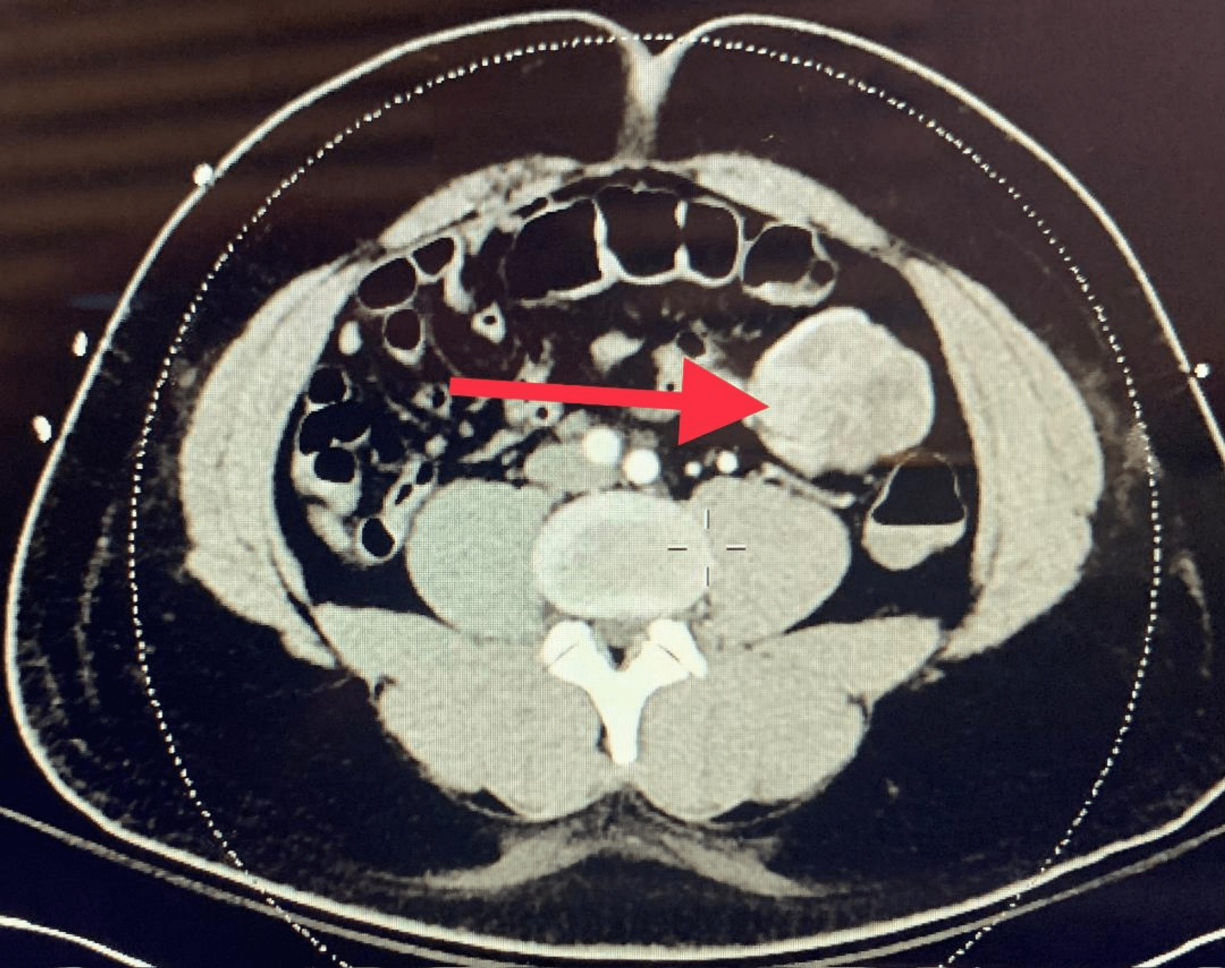
CT (axial) of the abdomen Soft tissue mass associated with small bowel loop in the left lower quadrant (red arrow) CT: computed tomography

A 33-year-old man with no contributory past medical history presented to the emergency room after a syncope episode. The patient was roused by his friend and felt lightheaded and collapsed again as he was going to the bathroom. Emergency medical services were called and reported that the patient had bloody diarrhea en route to the emergency room. Laboratory findings were within the normal range except for white blood cell (WBC) (15.5×10^3^/UL; reference range: 4.2-10.8×10^3^/UL) and hemoglobin (Hb) (11.8 g/dl; reference range: 14-18 g/dl). Syncope workup (brain scan, carotid duplex, echocardiogram) was within normal limits. Upper gastrointestinal endoscopy revealed a hiatal hernia, antral gastritis, and duodenal ulcer with no signs of active bleeding. Lower gastrointestinal endoscopy revealed a significant amount of fresh blood in the left colon with no active source. The following day, the patient had a precipitous drop of Hb (5.2 g/dl) associated with fresh bleeding per rectum requiring a massive transfusion. Computed tomography angiography (CTA) of the abdomen revealed no active extravasation of contrast and soft tissue mass associated with small bowel loops in the left lower quadrant measuring up to 5.1×5.6 mm concerning for GIST (Figure 1 and Figure 2).

**Figure 3:**
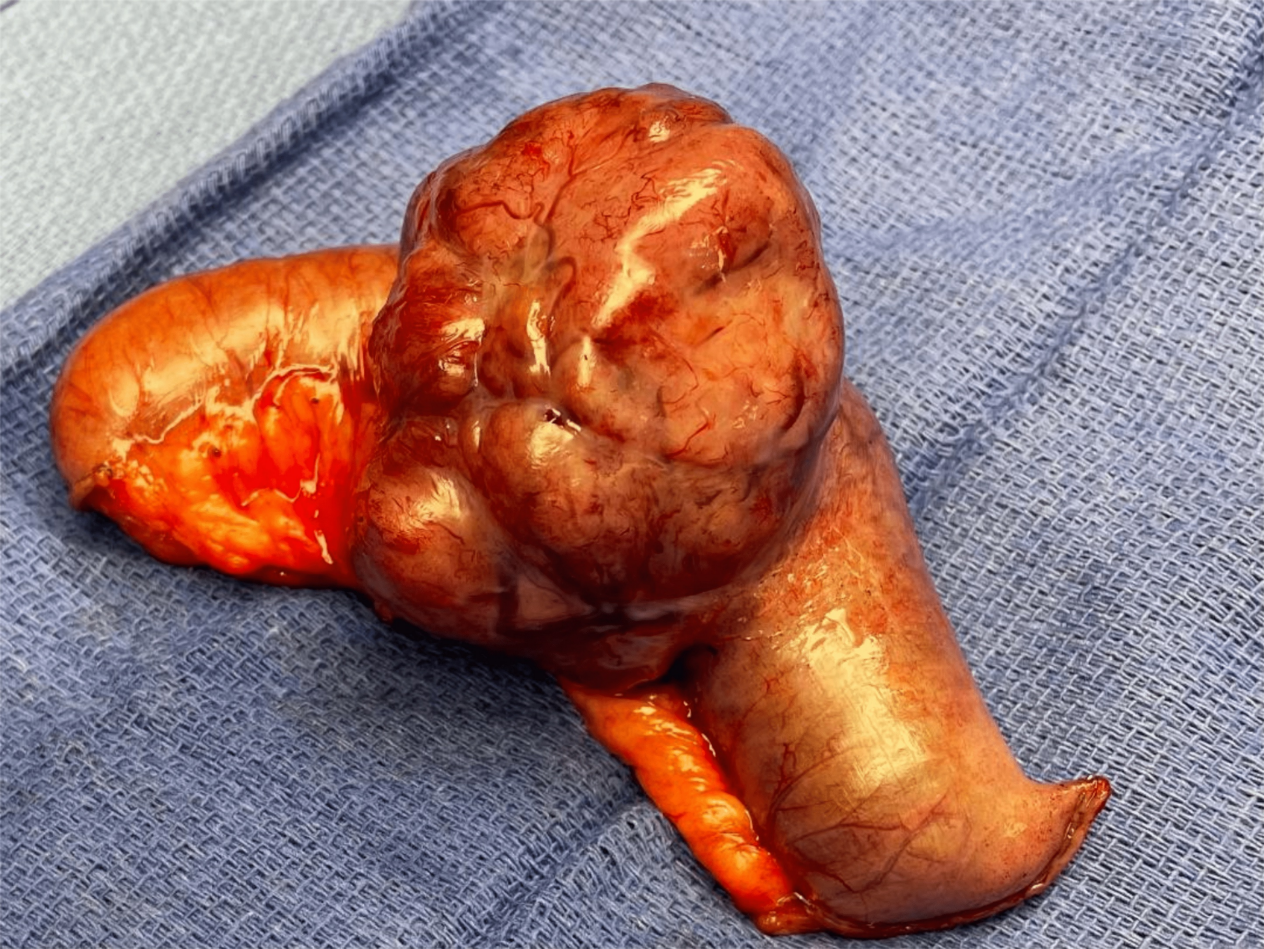
Resected small bowel loop The mass was seen on CT CT: computed tomography

The patient was taken to the operating room, and resection of the loop of the small bowel containing the mass was performed (Figure 3).

Histopathology confirmed GIST. The patient did well and was discharged from the hospital on postoperative day 3.

## Discussion

In 1983, Mazur and Clark introduced GISTs as a separate entity from gastrointestinal smooth muscle tumors [5]. The estimated unadjusted incidence is around 1/100,000/year [6]. Most patients present with nonspecific symptoms, such as bloating or early satiety, and are often discovered incidentally, most commonly in the stomach, followed by the small intestine [7]. Asymptomatic tumors less than 2 cm can be managed conservatively, and removal is recommended for all GISTs with a size ≥2 cm based on malignancy risk stratification by Miettinen and Lasota [8]. Tumor size, aggressiveness, and location dictate the clinical symptoms of GIST, ranging from abdominal pain, bowel obstruction, or gastrointestinal bleeding [9]. Life-threatening gastrointestinal bleeding can result from ulceration and necrosis of the overlying mucosa, along with submucosal destruction and invasion of nutrient vessels by the tumor. This is typically seen in aggressive GISTs, characterized by a mitotic index of ≥5 per 50 high-power fields and a tumor size greater than 4 cm [10]. CT with intravenous contrast is the most commonly used diagnostic modality for identifying stromal tumors. Endoscopic ultrasound is another valuable diagnostic tool, often revealing a hypoechoic mass in the early stages. As the GIST enlarges and invades surrounding structures, it may appear as a cystic or necrotic mass [11]. GISTs presenting with acute gastrointestinal bleeding require urgent surgical or endoscopic intervention or transcatheter arterial embolization [12,13]. Imatinib mesylate is a tyrosine kinase inhibitor used for controlling locally advanced and metastatic GIST and has been used as neoadjuvant and adjuvant drug therapy [14].

## Conclusions

Massive gastrointestinal bleeding secondary to GISTs is rare, diagnosed with contrast-enhanced CT. Depending on the location, bleeding can be controlled by endoscopic sclerotherapy, angioembolization, and/or emergent surgery. 
